# Short-term Response of Serum Cartilage Oligomeric Matrix Protein to Different Types of Impact Loading Under Normal and Artificial Gravity

**DOI:** 10.3389/fphys.2020.01032

**Published:** 2020-08-31

**Authors:** Maren Dreiner, Steffen Willwacher, Andreas Kramer, Jakob Kümmel, Timo Frett, Frank Zaucke, Anna-Maria Liphardt, Markus Gruber, Anja Niehoff

**Affiliations:** ^1^Institute of Biomechanics and Orthopaedics, German Sport University Cologne, Cologne, Germany; ^2^Human Performance Research Centre, Department of Sport Science, University of Konstanz, Konstanz, Germany; ^3^Institute of Aerospace Medicine, German Aerospace Center (DLR), Cologne, Germany; ^4^Dr. Rolf M. Schwiete Research Unit for Osteoarthritis, Orthopaedic University Hospital Friedrichsheim gGmbH, Frankfurt/Main, Germany; ^5^Department of Internal Medicine 3, Rheumatology and Immunology, Friedrich-Alexander-University Erlangen-Nuremberg, University Hospital Erlangen, Erlangen, Germany; ^6^Cologne Center for Musculoskeletal Biomechanics (CCMB), Faculty of Medicine, University of Cologne, Cologne, Germany

**Keywords:** articular cartilage, cartilage oligomeric matrix protein, artificial gravity, impact loading, countermeasure, centrifuge, reactive jumping

## Abstract

Microgravity during long-term space flights induces degeneration of articular cartilage. Artificial gravity through centrifugation combined with exercise has been suggested as a potential countermeasure for musculoskeletal degeneration. The purpose of this study was to investigate the effect of different types of impact loading under normal and artificial gravity conditions on serum concentrations of cartilage oligomeric matrix protein (COMP), a biomarker of cartilage metabolism. Fifteen healthy male adults (26 ± 4 years, 181 ± 4 cm, 77 ± 6 kg) performed four different 30-min impact loading protocols on four experimental days: jumping with artificial gravity elicited by centrifugation in a short-arm centrifuge (AGJ), jumping with artificial gravity generated by low-pressure cylinders in a sledge jump system (SJS), vertical jumping under Earth gravity (EGJ), and running under Earth gravity (RUN). Five blood samples per protocol were taken: 30 min before, immediately before, immediately after, 30 min after, and 60 min after impact loading. Serum COMP concentrations were analyzed in these samples. During the impact exercises, ground reaction forces were recorded. Peak ground reaction forces were significantly different between the three jumping protocols (*p* < 0.001), increasing from AGJ (14 N/kg) to SJS (22 N/kg) to EGJ (29 N/kg) but were similar in RUN (22 N/kg) compared to SJS. The serum COMP concentration was increased (*p* < 0.001) immediately after all loading protocols, and then decreased (*p* < 0.001) at 30 min post-exercise compared to immediately after the exercise. Jumping and running under Earth gravity (EGJ and RUN) resulted in a significantly higher (*p* < 0.05) increase of serum COMP levels 30 min after impact loading compared to the impact loading under artificial gravity (RUN +30%, EGJ +20%, AGJ +17%, and SJS +13% compared to baseline). In conclusion, both the amplitude and the number of the impacts contribute to inducing higher COMP responses and are therefore likely important factors affecting cartilage metabolism. RUN had the largest effect on serum COMP concentration, presumably due to the high number of impacts, which was 10 times higher than for the jump modalities. Future studies should aim at establishing a dose-response relationship for different types of exercise using comparable amounts of impacts.

## Introduction

A challenge of long-duration spaceflight is the degeneration of skeletal muscle and bone tissue (LeBlanc et al., 2007). After spaceflight, the most significant degenerative effects were observed for those limbs that normally have to sustain frequent impact loading and that work against Earth’s gravitational forces. For example, a stay on the International Space Station (ISS) for 6 months led to musculoskeletal degeneration of the lower limbs, even though exercise programs were carried out (Trappe et al., 2009). In particular, decrements of calf muscle peak power, force-velocity characteristics, and muscle fiber structure were observed (Trappe et al., 2009). Further, the reduced mechanical loading of the skeletal system in microgravity leads to bone loss, which is associated with a higher fracture risk (LeBlanc et al., 2007).

The effects of microgravity on human articular cartilage are not well-known, even though it is well-established that articular cartilage health and maintenance is highly dependent on an appropriate mechanical loading environment (Cattano et al., 2017). As a bradytrophic tissue with a slow metabolism and limited capacity for regeneration, cartilage is highly sensitive to disuse. Unloading and immobilization in both animal and human studies have caused cartilage thinning and softening (Haapala et al., 2000; Hudelmaier et al., 2006). Furthermore, weight-bearing articular cartilage of mice shows degradative changes in response to microgravity (Fitzgerald et al., 2019). Moreover, recent studies in humans have revealed that bed rest (Liphardt et al., 2009, 2016, 2018) and microgravity (Niehoff et al., 2016) may initiate catabolic processes indicated by serum biomarkers of articular cartilage metabolism.

Serum biomarkers monitor changes in cartilage metabolism (Bauer et al., 2006) and might have the potential to identify pathomechanical joint loading in order to detect osteoarthritis at early stage before irreversible damages occur (Kraus et al., 2010). One established biomarker, which reflects the current state of articular cartilage metabolism, is the cartilage oligomeric matrix protein (COMP). COMP consists of five identical glycoprotein subunits and is an extracellular cartilage matrix protein, interacting with collagen molecules to maintain the network (Halász et al., 2007). Enhanced serum COMP levels have been reported in different joint diseases, like osteoarthritis (Petersson et al., 1998; Clark et al., 1999), rheumatoid arthritis (Lindqvist et al., 2005; Wisłowska and Jabłońska, 2005), and knee injuries (Dahlberg et al., 1994), but also in response to physical activity (Pruksakorn et al., 2013; Cattano et al., 2017; Roberts et al., 2018). For example, serum COMP concentration rises due to cyclic exercise after 30 min of walking (Mündermann et al., 2005). COMP concentration rises with increasing movement speed (Denning et al., 2016) and distance (Neidhart et al., 2000; Kim et al., 2009), and also the type of exercise affects serum COMP concentration (Niehoff et al., 2011). Overall, these findings suggest that the serum COMP concentration is sensitive to magnitude, frequency, duration, and type of mechanical loading of the lower extremities.

Serum COMP concentration potentially reflects the extrusion of COMP fragments from the loaded articular cartilage (Niehoff et al., 2010). This mechanism could explain the decrease of serum COMP concentration during unloading and the increase after reloading of the lower extremities. Fourteen days of bed rest, an analog that can simulate some of the adaptational processes of space flight, lead to an average reduction of serum COMP level of −14.8% that recovered to baseline levels after being mobile again. In the same study, cartilage thickness decreased by 8.3% in the loaded region of the tibia compared from before to after bed rest (Liphardt et al., 2009).

Currently implemented exercise countermeasures for crewmembers of the ISS seem insufficient for long-term space travels such as missions to Mars (Horneck and Comet, 2006) and articular cartilage health has to date not been subject when tailoring exercise countermeasures. While the replacement of the iRED (interim resistive exercise device) with the ARED (advanced resistive exercise device) lead to reduced bone loss (Sibonga et al., 2015) as well as increased muscle strength in the trunk and lower limbs (English et al., 2015) of the ISS crew members. However, the size of the ARED makes it unlikely for it to be transported in a manned Mars mission (Konda et al., 2019). For example, NASA’s Orion vehicle currently has a planned habitable volume of around 9 m^3^, in comparison to 388 m^3^ on the ISS (Scott et al., 2019). In addition, at present, crew members of the ISS spend approximately 2.5 h per day exercising, including setup, stow, and personal hygiene, so the actual time of exercising is about 1.5 h (Petersen et al., 2016), making the training highly time-consuming (Coolahan et al., 2004; Buckey, 2006).

Consequently, exercise countermeasure research is ongoing to optimize devices and protocols for future space missions. One approach is the use of artificial gravity to mimic the Earth’s gravity of 1 *g* or even higher acceleration levels during flight. It has been suggested that already short periods of artificial gravity are sufficient to maintain musculoskeletal health (Clément et al., 2015). For instance, 1 h of daily centrifugation (2.5 *g* at the height of the feet) was sufficient to prevent muscle protein breakdown during 21 days of bed rest (Symons et al., 2009). However, in a complementary bed rest study, bone degeneration was not prevented, and a decrease in both bone mineral density and content was observed (Smith et al., 2009). These results may indicate that centrifugation alone is not a sufficient countermeasure for musculoskeletal degeneration in microgravity.

Exercise countermeasures can be designed so that multiple body compartments are affected (Shackelford et al., 2004). For example, jump training can prevent the musculoskeletal and cardiovascular system from deconditioning and has been recommended as a very time-efficient and effective type of countermeasure during space flight (Kramer et al., 2017a,b, 2018; Gruber et al., 2019). Reactive jumps are characterized by high ground reaction forces, high rates of force development, short ground contact times, and high leg stiffness (Kramer et al., 2012b), thus providing a high osteogenic stimulus (Ebben et al., 2010). In addition, reactive jumps are highly dynamic movements that require well-timed activation and coordination of muscles, thus providing a stimulus for the neuromuscular system, promoting the integration of several sensory inputs, such a muscle spindles, Golgi tendon organs, or visual and vestibular feedback (Gollhofer et al., 1987; Gollhofer and Kyröläinen, 1991; Dietz et al., 1992). Parameters such as the knee extensor torque as well as quadriceps strength have been shown to improve after 14-week of plyometric training (Chmielewski et al., 2016). In summary, combining artificial gravity with reactive jumping, a high-impact exercise mode with high ground reactions forces and high rates of force development that have been shown to be effective stimuli for maintaining bone, muscle, and cardiovascular health, might be a particularly suitable exercise mode to counteract musculoskeletal deconditioning during prolonged stays in microgravity.

The purpose of the present study was to analyze the acute response of serum COMP concentrations to four different high-impact exercises under normal and artificial gravity conditions to verify if artificial gravity in combination with reactive jumping is a suitable loading protocol to stimulate articular cartilage metabolism.

## Materials and Methods

### Participants

Fifteen healthy male participants were included in this crossover study. The participants were limited to male volunteers to gain a sufficiently homogenous group, especially because serum COMP concentration is sex-dependent (Jordan et al., 2003) and most of the previous studies in this area of research have focused on males (Cattano et al., 2017). Demographic data are summarized in Table 1. Inclusion criteria were an age between 20 and 35 years at the time of recruitment, good physical condition (2–3 training sessions per week), and no prior use of the countermeasure devices. Exclusion criteria were musculoskeletal disorders, acute or chronic injuries of the lower extremities, and knee surgery. The experimental protocol was approved by the Local Ethics Committee of the North Rhine Medical Association (Aerztekammer Nordrhein) and registered in the German Clinical Trial Registry (DRKS-ID: DRKS00014001; title: Reactive jump training under hypergravity – comparability of moments and effects on the metabolism of joint cartilage). Note that the same participants took part in an additional day of centrifugation in the context of the same study, the data of which have been published in Kramer et al. (2020). The data were not included in our analysis because the stimulus was irrelevant to our research question. Following the Declaration of Helsinki, informed written consent was obtained from all participants prior to their participation.

**Table 1:** Demographic participants characteristics (mean and 95% CI).

*N*	Age (years)	Body height (cm)	Body mass (kg)	BMI (kg/m^2^)
15	26 (24–29)	181 (177–185)	77.2 (74.0–80.5)	23.6 (22.7–24.6)

### Experimental Protocol

**Figure 1:**
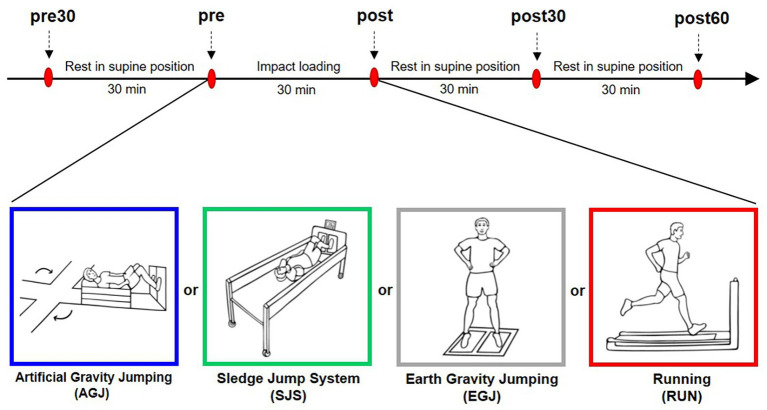
Experimental design. Red ovals denote the blood sampling time points once every 30 min. Before and after the impact loading the participants were in supine position. The impact loading changed on each experimental day. The four modes of impact loading are illustrated beneath the timeline (from left to right): jumping inside the short-arm-centrifuge (AGJ), jumping inside the sledge jump system (SJS), jumping without device (EGJ), and running on a treadmill (RUN).

On four experimental days, the participants performed four different impact loading exercise protocols with a duration of 30-min each. Except for the type of impact loading, the study protocol was identical on all four experimental days. The experimental days were separated by at least 3 days of rest and were carried out in random order but running was always the last protocol for organizational reasons. On the experimental days, participants arrived at the laboratory at roughly the same time of day. Then, an indwelling venous catheter was inserted into one cubital fossa. Subsequently, the necessary equipment (safety belt, pulsoxymeter, electrocardiogram, and blood pressure monitor) was attached to the participants, depending on the requirements of the loading condition. As a warm-up, the participants performed 10 squats and 10 heel raises. Afterwards, the first blood sample (pre30) was taken (see Figure 1), followed by 30 min of supine rest to reduce potential influences of preceding physical activity on the biomarker concentration (Mündermann et al., 2005). After the supine rest, the second blood sample was taken (pre). Subsequently, the participants completed the respective 30 min impact loading exercise with a duration of 30 min, followed by a blood sampling immediately after the exercise (post), again in a supine position. The participants remained supine until two more blood samples were collected after 30 min (post30) and 60 min (post60).

### Impact Loading

**Figure 2:**
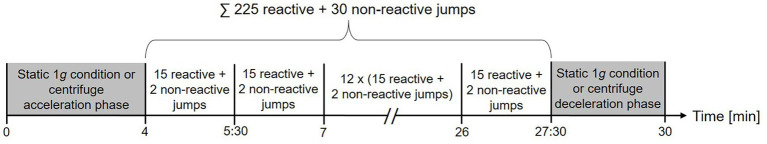
Timeline of the three jumping protocols. To make the three protocols comparable, we adhered to the schedule set by the centrifuge protocol: 4 min of getting the centrifuge ready and set to the correct g-level, followed by 15 series of 15 jumps each and a deceleration period of 2 min 30 s at the end. One series of jumps lasted 1 min 30 s, consisting of approximately 12 s of jumping, followed by approximately 75 s of rest.

The four different impact loading protocols with a duration of 30 min each were jumping under artificial gravity on the centrifuge (AGJ), jumping under artificial gravity on the sledge jump system (SJS), normal jumping under Earth gravity (EGJ), and running under Earth gravity (RUN). During three of these exercise modes (AGJ, SJS, and EGJ), the participants completed 15 × 15 reactive jumps with the instruction to jump as high as possible while keeping the ground contact time as short as possible. These 15 × 15 jumps were performed based on a fixed schedule, see Figure 2. For each series of 15 reactive jumps, the participants required approximately 12 s; short breaks were included in between series, taking around 75 s each. The total duration of each of the jump exercise modes was 30 min, and approximately 3 min of these 30 min were spent exercising.

#### Centrifuge

**Figure 3:**
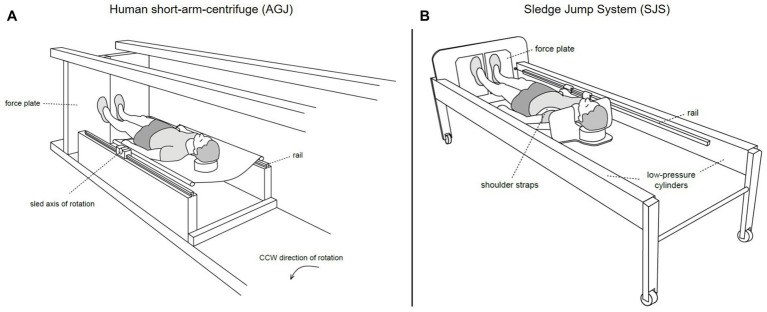
Schematic representations of the two devices which were used to simulate Earth gravity (Kramer et al., 2020). On the left **(A)**, illustration of the human short-arm-centrifuge (AGJ), horizontal force (artificial gravity) is created by the centrifugal force set with the rotation rate of the centrifuge. On the right **(B)**, illustration of the sledge jump system (SJS), horizontal force is created by low-pressure cylinders mounted inside the frame.

The artificial gravity jumping exercise (AGJ) was performed in a short-arm human centrifuge (for details, see Frett et al., 2014) located in the envihab research facility at the German Aerospace Center (DLR e.V.), Cologne, Germany, see Figure 3A. The centrifuge was constructed by AMST-Systemtechnik GmbH (Ranshofen, Austria) on behalf of DLR. At the end of the centrifuge arm, two force plates (OR6 Series, AMTI®, Watertown, MA, USA) were mounted and recorded ground reaction forces at a sampling frequency of 1,000 Hz.

The participants were placed in a supine position inside the centrifuge from the first blood sampling (pre30) until the last blood sampling (post60). The centrifuge rotated for 30 min counterclockwise during the jumping period. The rotational speed was adjusted according to the participant’s body weight. For example, the centrifuge’s rotational speed for a participant with a body weight of 700 N was set to a value that resulted in a summed ground reaction force of 700 N, i.e., an acceleration equivalent of 1 *g*. After the 1 *g* level was achieved, the participants performed 15 × 15 reactive jumps as described above.

#### Sledge Jump System

The second impact exercise mode was performed in a horizontal position on a sledge jump system (Novotec Medical GmbH, Pforzheim, Germany, see Figure 3B).

The SJS consists of a frame mounted on wheels and a lightweight sledge attached to rails (for details, see Kramer et al., 2017b). The artificial gravity is generated by two low-pressure cylinders, which pull the sledge toward two force plates (Leonardo®, Novotec Medical GmbH, Pforzheim, Germany) mounted at the bottom end of the SJS. The force generated by the low-pressure cylinders was adjusted to the participant’s body weight, resulting in an acceleration of 1 *g*.

During the 30-min exercise period, the participants performed 15 × 15 reactive jumps inside the SJS. Thereby the participants were fixed to the sledge with two straps around the shoulders. The SJS allows horizontal movement along the direction of the rails as well as rotational motions around a mediolateral axis. In this set-up, almost natural jumps were possible, as has been shown in previous studies (Kramer et al., 2010, 2012a,b). The participants performed the jumps with bare feet. The ground reaction force was recorded with two separated force plates for each foot (sampling frequency: 10,000 Hz).

#### Earth Gravity Jumping

Data from jumps conducted without any additional device under natural Earth gravity (EGJ) were examined as well. During the 30-min exercise period, the participants completed 15 × 15 vertical reactive jumps with bare feet and in an upright position. Two force plates (OR6 Series, AMTI®, Watertown, MA, USA) recorded the ground reaction forces during the jumps separately for each foot, with a sampling frequency of 1,000 Hz.

#### Running

A 30-min treadmill run (RUN) was performed for comparison with previous studies, as serum COMP concentration has been demonstrated to be sensitive to 30 min of running (Mündermann et al., 2009; Niehoff et al., 2010, 2011; Denning et al., 2016).

For the running (RUN) impact exercise, the participants were instructed to continuously run at a set speed of 2.2 m/s (i.e., about 8 km/h) on a motorized, force-instrumented treadmill (Treadmetrix, Park City, UT, USA) for 30 min. This set speed of 2.2 m/s was chosen for better comparability to previous studies (Niehoff et al., 2011; Firner et al., 2018). All participants wore the same cushioned running shoes (Brooks Glycerin) because the surface of the treadmill made barefoot running impracticable. Ground reaction force data were collected using four load cells (MC3A-500, AMTI®, Watertown, MA, USA) integrated into the treadmill, with a sampling frequency of 1,000 Hz. Ground reaction force data were recorded of 20 consecutive ground contacts each at six time points; after 3, 8, 13, 18, 23, and 28 min of running.

### Biomechanical Data Processing

The maximum of the horizontal (AGJ and SJS) or vertical (EGJ and RUN) component of the ground reaction force was determined and normalized to body mass using a custom Matlab code (R2015a, The MathWorks, Natick, MA, USA). Within the same code, the number of jumps were determined. For each subject and exercise mode, the mean value of the peak forces and the number of impacts were calculated, in general 15 reactive jumps plus two non-reactive jumps (at the beginning and end of each series) per series, resulting in a total amount of 255 impacts. Due to the appearance of incorrect strikes (e.g., contact of one foot on both force plates) onto the force plate, a trimmed mean value (20% trimmed mean) was used for averaging the peak force data to exclude outliers within the jumping exercise protocols.

The peak force data were averaged over all analyzed ground contacts in the RUN protocol and the total number of ground contacts were estimated by multiplying the run duration (30 min) with the average step frequency calculated over the six measurement time points, for each participant.

### Serum COMP Concentration Analysis

Blood samples (each 8 ml) were drawn from the antecubital vein using indwelling venous catheters (Vasofix® Sadty, B. Braun, Melsungen, Germany) in combination with vacutainers (BD Vacutainer® SSTTM II Advance, Becton Dickinson and Co., Franklin Lakes, NJ, USA). Blood samples were allowed to clot for 30 min; blood serum was isolated after centrifugation (1,500 rcv for 10 min), aliquoted into freezing tubes, and stored at −80°C until analysis.

Serum COMP concentrations were determined using a commercial ELISA (COMP® ELISA, AnaMar AB, Göteborg, Sweden) and following manufacturer’s instructions. The plate was coated with anti-COMP (mouse monoclonal antibodies). As enzyme conjugate, peroxidase-anti-COMP (mouse monoclonal antibodies ∼10 μg/ml) was used and later for detection purposes enzyme substrate 3,3',5,5'-teramethylbenzidin was added. The reaction was then stopped by 0.5 M H_2_SO_4_, and the light absorbance of the solution was read at 450 nm with the aid of a plate reader (TriStar2 LB 942 Modular Multimode Microplate Reader, Berthold Technologies, Bad Wildbad, Germany). The measured optical densities were then converted into the serum biomarker concentrations applying a four-parametric logistic regression in SigmaPlot 8.0 (Systat Software Inc., San Jose, CA).

For the analyses, the investigators were blinded to the impact loading protocols and time points at which the samples were taken, and all samples were analyzed in duplicate and random order. The detection limit of the COMP ELISA was <0.1 U/L and the inter‐ and intra-assay coefficients of variation were < 5% according to the manufactures specifications. To minimize the inter-assay variability, all samples of one subject were analyzed on the same plate.

### Statistics

Statistical analyses were performed using Statistica 7.1 (StatSoft GmbH, Hamburg, Germany). The normal distribution of the variables was tested with the Kolmogorov-Smirnov test and Mauchly’s test for sphericity was done. If the sphericity assumption was violated, we used Greenhouse-Geisser corrections.

For the statistical analysis of the kinetic data, a student’s *t* test for dependent samples was applied to identify differences between left and right leg. A one-way (factor: impact loading) analysis of variance (ANOVA) was performed for repeated measurements on the “peak force” and “peak force x ground contacts” values. For the identification of pairwise differences between the impact loadings, Fisher’s least significant difference (LSD) test was used.

Cartilage biomarker data measured before the impact loading (“pre30” and “pre”) were analyzed using the student’s *t* test for dependent samples. A two-way (factors: loading protocols and time point) analysis of variance (ANOVA) for repeated measures and Duncan’s multiple range test for *post hoc* analysis were performed to detect differences between the serum biomarker levels (except “pre30” blood sampling).

All variables were described as mean values and 95% confidence intervals. Statistical tests were performed on absolute values, and only for graphical presentation biochemical data were normalized to baseline values at the blood sampling time point “pre”. The level of significance was set at *α* < 0.05 for all statistical tests.

## Results

### Peak Forces

Biomechanical loading was assessed *via* peak ground reaction forces and the number of impacts. Due to technical problems, the recording of the ground reaction forces failed for one participant during the RUN exercise. Therefore, this participant was not considered for further analyses, resulting in an effective sample of *N* = 14 for the peak force analyses.

Mean peak forces were not significantly different on the left compared to the right side for all exercise modalities. Therefore, the mean value of left and right peak forces was computed and used for further analyses.

**Figure 4:**
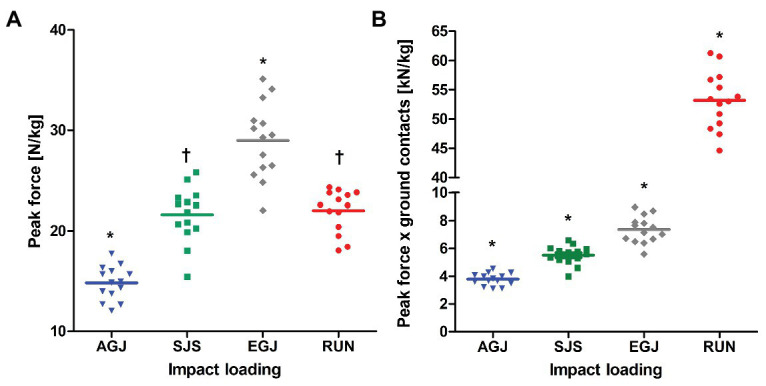
Mean (lines) and individual (marks) of peak forces [N/kg] **(A)** and peak force*ground contacts [kN/kg] **(B)** per leg, normalized to body mass for the four types of impact loading (artificial gravity elicited by centrifugation AGJ, artificial gravity in the sledge jump system SJS, normal vertical jumps EGJ and running on a treadmill RUN (*N* = 14)). An asterisk symbol (*) denotes a significant difference compared to all other types of impact loading (*p* < 0.001), and a ^†^ symbol a significant difference compared to AGJ and EGJ (*p* < 0.001).

Averaged peak forces were different between loading conditions and increased from AGJ to SJS to EGJ (*p* < 0.001, Figure 4A). The RUN exercise had (*p* < 0.001) higher mean peak forces compared to the AGJ exercise on the centrifuge and lower (*p* < 0.001) mean peak forces compared to the EGJ exercise. However, no significant difference (*p* = 0.615) could be identified between the RUN and the SJS exercise.

The number of impacts was very similar for all jumping exercises as they were predefined to 15 × 15 reactive jumps (255 impacts per leg) during the 30 min (see Figure 2). In comparison, the number of impacts was much higher for the RUN exercise (around 2,400 impacts per leg) compared to all jumping exercises (AGJ, SJS, and EGJ). Also, no rest periods were planned in the RUN protocol in contrast to the jumping exercises, i.e., 30 min of running are compared with effectively 3 min of jumping. The product of peak force and number of impacts was different between all four types of impact loading (*p* < 0.001), increasing from AGJ, SJS, EGJ to RUN (see Figure 4B).

### Serum COMP Concentration

**Figure 5:**
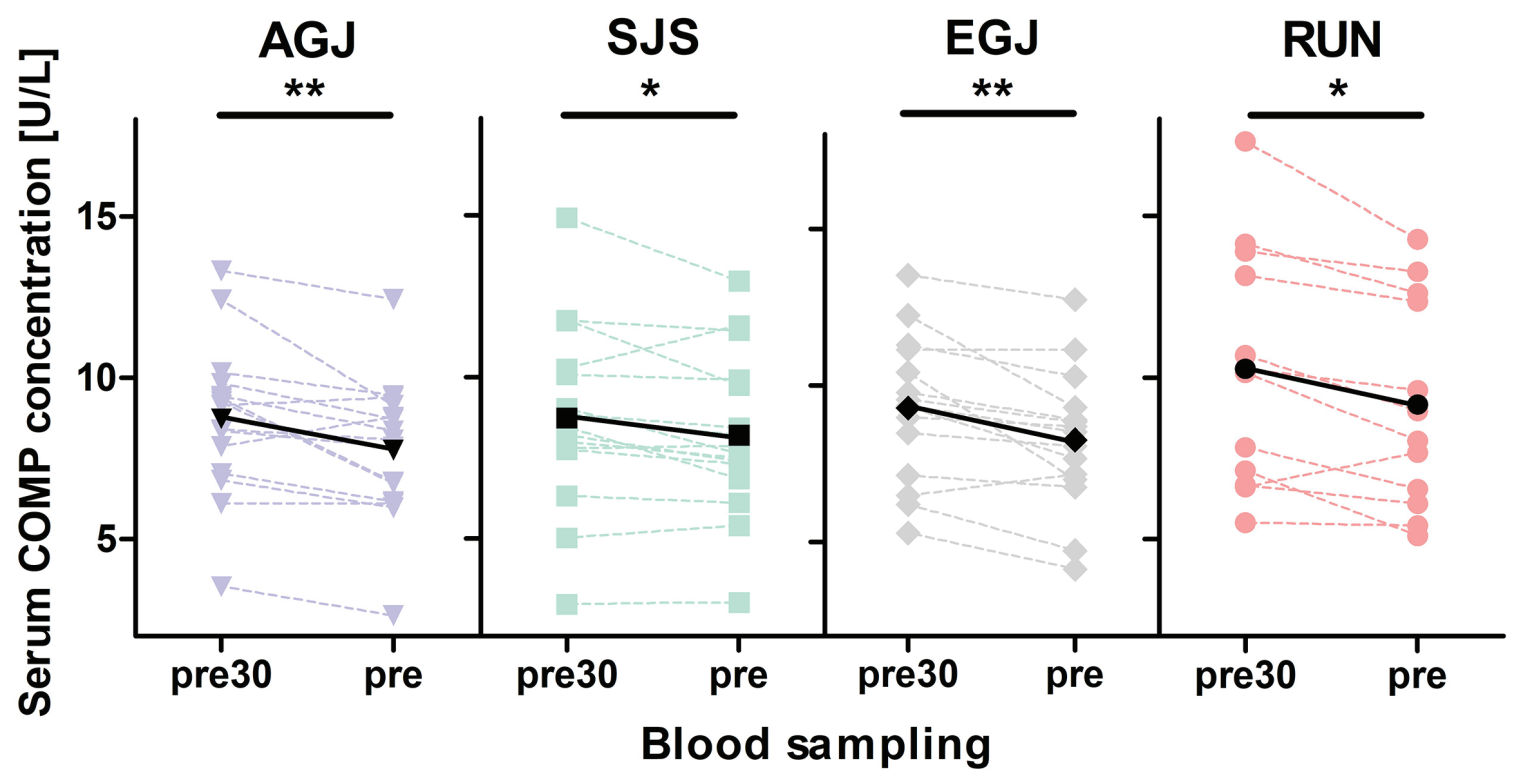
Mean (black marks) and individual (colored marks) of absolute serum COMP concentrations [U/L] before (pre30) and after (pre) 30 min of rest for the four different types of impact loading AGJ (*N* = 15), SJS (*N* = 15), EGJ (*N* = 15) and RUN (*N* = 12). ^*^Significantly (*p* < 0.05) different between the blood samplings “pre30” and “pre.” ^**^Significantly (*p* < 0.01) different between the blood samplings “pre30” and “pre.”

Blood sampling failed in three individuals for the first measurement (pre30) during the RUN protocol, due to problems with the indwelling catheter, which reduced the sample size to *N* = 12 for the pre30 measurement of this protocol. The mean serum COMP concentration decreased significantly from before (pre30) to after (pre) 30 min rest for all exercises (see Figure 5). Also, the pre serum COMP level of the RUN protocol was significantly higher (*p* < 0.05) compared to the other exercise protocols (AGJ, SJS, and EGJ).

**Figure 6:**
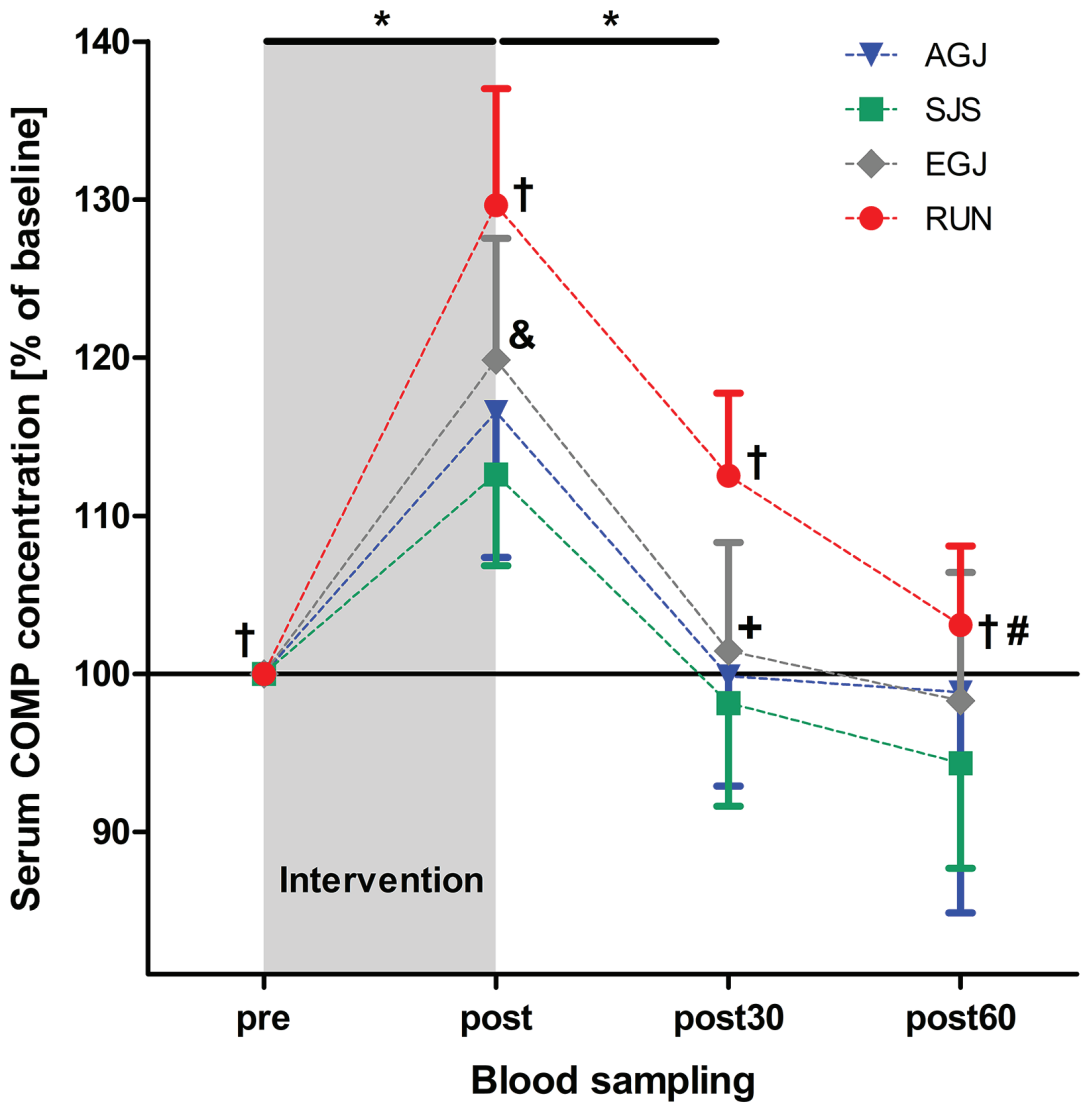
Mean (and 95% CI) serum COMP concentrations as percent of baseline values directly before (pre), immediately after (post), as well 30 min (post30) and 60 min (post60) after exercise for AGJ, SJS, EGJ, and RUN (*N* = 15). ^*^Significantly (*p* < 0.001) different compared to the preceding time point within the same loading protocol for AGJ, SJS, EGJ and RUN. ^#^Significantly (*p* < 0.001) different compared to the preceding time point for RUN within the same loading protocol. ^†^Significantly (*p* < 0.05) different compared to all other loading protocols for RUN within the same time point. ^&^Significantly (*p* < 0.05) different compared to all other loading protocols for EGJ within the same time point. ^+^Significantly (*p* < 0.05) different between the AGJ and the EGJ protocol within the same time point.

The mean serum COMP concentration increased significant (*p* < 0.001) immediately after all four types of impact loading (“pre” to “post,” see Table 2; Figure 6). This increase from “pre” to “post” was highest (+30%) for the RUN exercise and significantly (*p* < 0.05) different compared to all jumping exercises (AGJ, SJS, and EGJ). The second-highest increase (+20%) was identified for the EGJ exercise, which was significantly lower (*p* < 0.05) compared to RUN but significantly higher (*p* < 0.05) compared to the AGJ or SJS exercise. No significant difference between the AGJ and the SJS loading protocol could be detected immediately after the exercise. The increase in the serum COMP concentration compared to baseline was +17% for AGJ and +13% for SJS.

**Table 2:** Mean and 95% CI of absolute serum cartilage oligomeric matrix protein (COMP) concentrations [U/L] before and after the four types of impact loading: centrifuge (AGJ), sledge jump system (SJS), jumping (EGJ), and running (RUN).

Blood sampling	pre	post	post30	post60
Centrifuge (AGJ)	7.8 (6.5–9.0)	8.9 (7.6–10.2)^*^	7.8 (6.5–9.1)^*^	7.9 (6.4–9.3)
Sledge jump system (SJS)	8.2 (6.8–9.6)	9.2 (7.6–10.9)^*^	8.1 (6.4–9.7)^*^	7.8 (6.2–9.3)
Jumping (EGJ)	8.3 (7.0–9.5)	9.8 (8.3–11.4)^*^^,^^&^	8.5 (7.0–10.0)^*^^,^^+^	8.1 (6.8–9.5)
Running (RUN)	9.1 (7.4–10.7)^†^	11.5 (9.8–13.3)^*^^,^^†^	10.1 (8.4–11.7)^*^^,^^†^	9.2 (7.7–10.7)^†^^,^^#^

* Significantly (*p* < 0.001) different compared to the preceding time point within the same loading protocol for AGJ, SJS, EGJ, and RUN.

† Significantly (*p* < 0.05) different compared to all other loading protocols for RUN within the same time point.

& Significantly (*p* < 0.05) different compared to all other loading protocols for EGJ within the same time point.

# Significantly (*p* < 0.001) different compared to the preceding time point for RUN within the same loading protocol.

+ Significantly (*p* < 0.05) different between the AGJ and the EGJ protocol within the same time point.

Subsequently, from “post” to “post30” serum COMP concentration decreased significantly (*p* < 0.001) for all four exercise modes whereby the serum COMP concentration was still significantly higher for the running exercise (RUN) compared to all jumping exercises (AGJ, SJS, and EGJ; *p* < 0.05). In addition, the AGJ protocol leads to a significantly higher (*p* < 0.05) decrease in the serum COMP concentration compared to the EGJ jumping exercise.

Only for the RUN exercise, serum COMP significantly decreased from 30 to 60 min after the exercise (*p* < 0.001) but was still significantly (*p* < 0.05) higher in this group 60 min after the impact loading compared to the jumping exercises (AGJ, SJS, and EGJ). In contrast, the serum COMP level remained unchanged for the jumping exercises (AGJ, SJS, and EGJ) between 30 and 60 min after impact loading.

## Discussion

The purpose of the present study was to analyze the acute response of serum COMP concentration to four different high-impact exercises under normal and artificial gravity conditions to verify if artificial gravity in combination with reactive jumping is a suitable loading protocol to stimulate articular cartilage metabolism.

Serum COMP concentration increased significantly immediately after the AGJ and SJS impact loading and both potential countermeasures affected articular cartilage metabolism. However, serum COMP levels for both AGJ and SJS exercises did not reach those from the EGJ and RUN exercises, even though peak forces for SJS were comparable to the peak forces recorded during RUN, suggesting that the number of impacts also plays an important role for the response of the cartilage metabolism.

### Peak Forces

Significantly lower peak forces were observed during the AGJ compared to the other jumping protocols (SJS and EGJ). These lower peak forces are likely due to several challenges participants face when trying to adapt to the artificial gravity force field elicited by centrifugation: in addition to the unaccustomed horizontal body position in a system that restricts movement, the participant has to adapt to a non-constant force field as well as the presence of Coriolis forces. The force acting on an object during centrifugation depends on the object’s distance to the center of rotation, i.e., it will change during movement and it is higher for parts of the object that are further away from the center of rotation. The effects of these challenges on ground reaction forces have also been reported for other human centrifuges with different *g*-levels (Yang et al., 2007).

The peak forces measured on the SJS were similar to previously published values using the same device (Kramer et al., 2010, 2012a,b) and were significantly higher than on the centrifuge (AGJ) but significantly lower than during vertical jumping (EGJ). The lower peak forces on the SJS compared to the EGJ exercise have been proposed to be primarily a result of lacking familiarization as these differences between jumps in the SJS and normal jumps can be substantially reduced after some weeks of familiarization (Kramer et al., 2012a). Differences remaining after familiarization could be due to the horizontal body position as well as technical constraints of the SJS and the presence of gravito-inertial forces acting perpendicular to the movement direction (Kramer et al., 2010).

While for the AGJ and the SJS exercises, an increase of the peak force values from the first to the last jumping series could be observed, no increase was noticed for the EGJ exercise. This finding strengthens the notion that a learning effect on both the centrifuge and the SJS during the 15 jump series occurred, whereas the participants were familiar with the normal vertical jumps. Thus, increased peak forces and a more natural movement pattern would probably be achieved after several weeks of training on the SJS as well as on the centrifuge (Piotrowski et al., 2018).

Nevertheless, neither the AGJ nor the SJS impact loading elicited peak forces that were as high as the ones recorded during jumps in normal gravity conditions. It should be noted though that normal reactive jumps have been identified as the exercise mode that elicits the highest peak forces (Ebben et al., 2010), and that somewhat lower forces such as the ones observed for the jumps in the SJS or running on the treadmill are probably more than sufficient to elicit bone and cartilage adaptations, as demonstrated for bone mineral density and bone mineral content in a recent bed rest study (Kramer et al., 2017b). As proposed for bone in the mechanostat theory (Frost, 1987), there might be a certain threshold that mechanical loading has to exceed in order to maintain cartilage, but further research is required to confirm such a threshold concept and determine these thresholds for cartilage. It is also likely that not only the amplitude of the peak forces are important in this context, but also the number of impacts, as demonstrated by the differences in serum COMP levels of RUN vs. the jump exercise modes, which featured only about 255 impacts per leg (AGJ, SJS, and EGJ) compared to about 2,400 impacts per leg during the 30 min of continuous treadmill running (RUN).

### Serum COMP Concentration

The results of this study show similar ranges in serum COMP levels and a similar course of response (see Table 3) compared to previous studies that used a similar 30-min running exercise at around 2.2 m/s and the same ELISA kit for serum analysis (Niehoff et al., 2010, 2011; Firner et al., 2018). Furthermore, the significant decrease of COMP concentration from before (pre30) to after rest (pre) confirmed the relevance of the resting period to reach equilibrium in serum COMP before measuring the response to the exercise protocol.

**Table 3:** Comparison of mean serum COMP concentrations [U/L] and 95% CI or standard deviation, respectively, in-between studies with a 30 min running protocol, analyzed with the same commercial ELISA (AnaMar). Data and statistic taken from literature. “X” is indicating no available information.

Blood sampling	pre	post	post30	post60	Δpre-post (actual)	Δpre-post (percentage of baseline)
Running (RUN)	9.1 (7.4–10.7)	11.5 (9.8–13.3)^*^	10.1 (8.4–11.7)^*^	9.2 (7.7–10.7)^*^	2.5	+26%
Firner et al. (2018; passive orthesis)	7.5 (6.4–8.7)	9.8 (8.8–10.8)^*^	8.8 (7.5–10.1)^*^	8.7 (7.6–9.8)	2.3	+31%
Niehoff et al. (2011)	7.3 (5.6–8.9)	9.1 (7.2–11.0)^*^	8.6 (7.1–10.1)	7.9 (6.1–9.6)	1.8	+25%
Niehoff et al. (2010)	6.9 ± 1.7	9.5 ± X^*^	X	X	2.7	+38%

* Significantly (*p* < 0.05) different from preceding blood sampling.

Serum COMP levels increased for all types of impact loading in response to the applied exercise protocols. However, the underlying mechanistic processes causing the increase of the serum COMP concentration after physical activity and the physiological meaning of changes in serum COMP levels are not entirely understood. Hyldahl et al. (2016) suggested that the mechanical joint loading facilitates the diffusion of proteins, for example, COMP, from joint space to serum, causing a decrease in synovial fluid concentration and an increase in serum concentration. The efflux of COMP out of the synovium may contribute to articular cartilage health by maintaining the COMP to collagen ratio.

Another possible explanation for the increase of COMP concentration following physical exercise is that the changes in serum COMP levels may indicate a short-term adaption of the viscoelastic properties of the articular cartilage. COMP has been suggested to function as an adaptor protein, and in this role, it is considered to modulate extracellular matrix synthesis and tissue remodeling in different physiological conditions (Tan and Lawler, 2009; Agarwal et al., 2012).

High values of COMP measured in a standardized protocol with a sufficient resting period before drawing blood can be interpreted as an indicator for changes in extracellular matrix metabolism, but also as a risk factor for degenerative joint diseases like osteoarthritis (Garnero et al., 2001; Jiao et al., 2016; Mobasheri et al., 2017). Thus, when analyzing cartilage biomarker concentrations, permanent, temporary, or acute responses (e.g., after exercise) have to be distinguished in the interpretation of mechanosensitive cartilage biomarkers like COMP. In our study, in contrast to the clinical use of COMP as a biomarker of osteoarthritis, the acute response of COMP to mechanical loading was analyzed. Here, an increase in serum COMP concentration was interpreted in a positive way, such that the loading protocol was sufficient to stimulate cartilage metabolism. However, it is not known to date, at what magnitude and increase in serum COMP to an acute stimulus can indicate a positive adaptation or overload. In this context, it is noteworthy that before the start of the exercise, the serum COMP level in the RUN protocol was significantly higher (*p* < 0.05) compared to the other protocols. This difference could be due to seasonal variations of the resting COMP concentration. Increasing resting COMP levels were observed by Hoch et al. (2012) over the season for collegiate soccer athletes. In addition, it is recommended for the examination of bone biomarkers that the seasonal variants should be considered (Roberts et al., 2019). Most of the participants in this study were sports students, and the first three measurements (AGJ, SJS, and EGJ) were performed during semester break, while the data of the RUN protocol were collected during semester when the general physical activity could be considered higher because of practical courses. Potentially this generally leads to an increase of the serum COMP concentrations despite the exercise constraints around the data collection time points.

Interestingly, even with higher resting values (pre) the RUN protocol showed higher serum COMP concentration immediately after exercise compared to all jumping exercise modalities (AGJ, SJS, and EGJ). Further, for the time point “post,” a significant difference in COMP level for the EGJ compared to the horizontal jumping exercises (AGJ and SJS) was detected. Very likely this is due to differences in loading characteristics used in the four impact loading protocols. As mentioned in the introduction, it has been suggested that the serum COMP concentration is related to magnitude, frequency, and type of mechanical loading of the lower extremities (Niehoff et al., 2011). For the EGJ exercise, significantly higher peak forces (“amplitude”) were detected compared to the horizontal jumping exercises (AGJ and SJS). This may indicate that jumping in “normal” *g*-conditions has a higher effect on serum COMP levels than jumping with artificial gravity.

In the RUN protocol, peak forces were lower than during normal vertical jumping, but serum COMP levels were significantly higher than during any of the jumping protocols. In contrast to the jump exercise protocols, the RUN protocol did not include any breaks and was therefore marked by a much higher number of impacts compared to the jump protocols (by a factor of almost 10). This finding indicates that (a) both the magnitude of the impact loading and the number of impacts influence serum COMP levels and (b) the relationship between the number of impacts and their effect on serum COMP levels is probably not a linear one, otherwise a 10-fold increase in the number of impacts with similar peak forces (RUN vs. SJS) would have resulted in a 10-fold difference in the increase in COMP levels, but only a 1.3-fold increase was observed. A study with a systematic variation of peak forces and number of impacts could be conducted in the future to investigate this matter in greater detail. In addition, one has to consider that the range of motion in the joint is different between the exercise types.

Several studies have detected that cyclic loading at a physiological level has an anabolic effect on chondrocyte metabolism compared to static loading, which is more likely to initiate catabolic processes (Palmoski and Brandt, 1984; Mouw et al., 2007; Sharma et al., 2007; Ng et al., 2009; Bleuel et al., 2015). It has also been shown in engineered articular cartilage that dynamic compression could increase COMP concentration and the concentration of its binding partners, collagen type II and IX, as well as the glycosaminoglycan content (Ng et al., 2009; Pingguan-Murphy and Nawi, 2012). The increase in COMP concentrations due to dynamic mechanical loading improves the cell matrix interactions, since the COMP molecule has five subunits, which can potentially bind to five collagen molecules, thus bringing them to close proximity (Heinegård, 2009).

Assuming that a high serum COMP concentration after dynamic physical exercise is beneficial for articular cartilage assembly, the RUN protocol would have the strongest positive effect on joint health compared to the three jumping exercise modalities. However, the loading characteristics between the different impact loading protocols are not identical and, thus, this effect could be explained by the much higher number of impacts used in the RUN protocol. Further investigation in this setting should aim at investigating a dose-response relationship using protocols matched for the number of impacts.

## Limitations

Some limitations should be kept in mind when interpreting the results of this study: With 15 participants the sample size was rather small, although the randomized cross-over design and standardization measures reduce the impact of inter-individual differences in the analyses. As the serum COMP concentration is sex-dependent (Jordan et al., 2003), we only included male volunteers to (1) be able to compare results with previous studies (Niehoff et al., 2010, 2011; Firner et al., 2018) and (2) increase the power of our small sample size. Furthermore, circulating serum biomarkers do not give information about the localization of increased/decreased cartilage metabolism. Additional magnetic resonance imaging (MRI) could have provided valuable information about the localization of cartilage adaptation after the different types of impact loading and, thus, complementing the COMP marker results with data about deformation and composition of cartilage. Finally, the transfer of results from Earth to a microgravity environment in space should be performed with caution until the data are confirmed with experiments on board of the ISS. The loading characteristics in microgravity compared to normal gravity are expected to be slightly different, depending on the way the gravitational force is substituted in a microgravity environment.

## Conclusion

In summary, the results of this study show that all four types of impact loading affected articular cartilage metabolism. However, jumps in artificial gravity conditions (AGJ and SJS) led to a lower increase in serum COMP concentrations compared to the reactive jumps in the vertical direction (EGJ) under normal gravitational conditions. Based solely on these results, a higher magnitude of mechanical loading during the artificial gravity condition should be pursued. This could be achieved by increasing jumping frequency or increasing the *g*-level.

Running on the treadmill (RUN) leads to the greatest increase in serum COMP concentration after the exercise, indicating that running could be a sufficient stimulus to counteract articular cartilage degeneration. However, this effect is very likely to be due to the fact that for RUN the number of impacts were 10 times higher than for the jump modalities. Thus, the frequency and magnitude of impact are very likely important factors as well as the range of motion in the respective joint. Future studies should aim at investigating a dose-response relationship for different types of exercise using comparable amounts of impacts.

## Data Availability Statement

The raw data supporting the conclusions of this article will be made available by the authors, without undue reservation.

## Ethics Statement

The studies involving human participants were reviewed and approved by Local Ethics Committee of the North Rhine Medical Association (Aerztekammer Nordrhein). The patients/participants provided their written informed consent to participate in this study.

## Author Contributions

AN, FZ, A-ML, JK, AK, and MG contributed to the conception and design of the study. MD, SW, JK, AK, TF, and AN collected the data. MD, SW, JK, AK, and AN analyzed the data. MD, JK, AK, AN, FZ, A-ML, and MG interpreted the results. MD and AN wrote the manuscript. All authors contributed to manuscript revision, read and approved the submitted version.

### Conflict of Interest

The authors declare that the research was conducted in the absence of any commercial or financial relationships that could be construed as a potential conflict of interest.

## Abbreviations

AGJ: Artifical gravity jumping (centrifuge)
COMP: Cartilage oligomeric matrix protein
DLR: Deutsches Zentrum für Luft‐und Raumfahrt (German Aerospace Center)
EGJ: Earth gravity jumping
ELISA: Enzyme-linked immunosorbent assay
ISS: International Space Station
RUN: Running
SJS: Sledge jump system
